# Treatment Outcomes in Patients with Internet Addiction: A Clinical Pilot Study on the Effects of a Cognitive-Behavioral Therapy Program

**DOI:** 10.1155/2014/425924

**Published:** 2014-07-01

**Authors:** K. Wölfling, M. E. Beutel, M. Dreier, K. W. Müller

**Affiliations:** Outpatient Clinic for Behavioral Addictions, Department for Psychosomatic Medicine and Psychotherapy, University Medical Center Mainz, Untere Zahlbacher Straße 8, 55131 Mainz, Germany

## Abstract

Internet addiction is regarded as a growing health concern in many parts of the world with prevalence rates of 1-2% in Europe and up to 7% in some Asian countries. Clinical research has demonstrated that Internet addiction is accompanied with loss of interests, decreased psychosocial functioning, social retreat, and heightened psychosocial distress. Specialized treatment programs are needed to face this problem that has recently been added to the appendix of the DSM-5. While there are numerous studies assessing clinical characteristics of patients with Internet addiction, the knowledge about the effectiveness of treatment programs is limited. Although a recent meta-analysis indicates that those programs show effects, more clinical studies are needed here. To add knowledge, we conducted a pilot study on the effects of a standardized cognitive-behavioral therapy program for IA. 42 male adults meeting criteria for Internet addiction were enrolled. Their IA-status, psychopathological symptoms, and perceived self-efficacy expectancy were assessed before and after the treatment. The results show that 70.3% of the patients finished the therapy regularly. After treatment symptoms of IA had decreased significantly. Psychopathological symptoms were reduced as well as associated psychosocial problems. The results of this pilot study emphasize findings from the only meta-analysis conducted so far.

## 1. Introduction

Numerous studies of the past decade point to Internet addictive behavior as a growing health issue in different parts of the population. Prevalence estimations range up to 6.7% within adolescents and young adults in southeast Asia [1], 0.6% in the United States [2], and between 1 and 2.1% in European countries [3, 4] with adolescents showing even increased prevalence rates (e.g., [4]). Based on these observations, the APA has decided to include Internet Gaming Disorder—one common subtype of Internet addiction (IA)—into section III of the DSM-5 “as a condition warranting more clinical research and experience before it might be considered for inclusion in the main book as a formal disorder” [5].

People affected by IA report symptoms resembling those known from substance-related and other nonsubstance-related (e.g., gambling disorder) addiction disorders. They display a strong preoccupation with Internet activities, feel an irresistible urge to go online, show increasing hours spent online (tolerance), feel irritated and dysphoric when their online access is restricted or denied (withdrawal), keep on going online despite negative consequences in different areas of life (e.g., conflicts with family members and decreasing achievements in school, college, or job), and are not able to cut back from their behavior (loss of control). Since further parallels have been reported regarding shared neurobiological features (e.g., [6]; for a review see [7]) and similarities in underlying personality traits (e.g., [8, 9]), it has been proposed to perceive IA as another type of nonsubstance-related addiction disorder. Furthermore, increased rates of comorbid IA within patients suffering from other forms of addiction that have been reported solidify this assumption [6, 10].

Clinical studies underpin increased psychopathological symptoms and decreased levels of functioning in patients [11], deteriorating quality of life [12], social retreat, and isolation, respectively [13], as well as high levels of psychosocial and psychopathological symptoms [14, 15]. For example, Morrison and Gore [16] reported high levels of depression within a sample of 1319 study participants. Likewise, Jang and colleagues [17] documented increased psychosocial strain, especially concerning obsessive-compulsive and depressive symptoms in adolescents suffering from IA.

Since IA is more and more recognized as a serious mental disorder causing distress and decreased levels of functioning in those affected by it, increasing efforts to develop and document different treatment strategies have emerged, including psychotherapeutic and psychopharmacological interventions for IA [18]. Although one has to admit that current clinical investigations are lacking in methodological quality or are based on comparably small patient samples (for a review of treatment outcome studies on IA see King et al. [18]), the first findings regarding response and remission after treatment in IA are promising.

One study that met several quality standards of clinical outcome studies according to the analytic review by King et al. [18] investigated effects of a multimodal cognitive-behavioral program in adolescents with IA [19]. 32 patients treated due to IA were statistically compared to a wait-list control group receiving no treatment (24 subjects). Primary endpoints of this study included a self-report measure for IA (Internet Overuse Self-Rating Scale by Cao and Su [20]) as well as self-report measures assessing time management skills and psychosocial symptoms. Changes in these outcome measures were assessed prior to, immediately after, and at the end of the treatment. A followup was performed six months after the treatment. The results showed that, in both groups, a significant decrease of IA-symptoms was observable and also stable over the period of six months. However, only the treatment group was displaying significant improvement in time management skills and decreasing psychosocial problems regarding lower anxiety and social problems.

Likewise, studies applying psychopharmacological treatment have demonstrated promising results indicating that patients with IA benefit from SSRI and methylphenidate [21, 22], matching findings from clinical evidence in the treatment of patients with gambling disorder [23].

Moreover, a recently published meta-analytic study by Winkler and colleagues [24] that included 16 clinical trials with different therapeutic approaches based on 670 patients indicates high effectiveness of treatment of IA: the detailed results suggest that there were significant differences depending on the type of therapeutic treatment with cognitive-behavioral programs displaying higher effect sizes (*d* = 0.84–2.13) regarding decreased symptoms of IA than other psychotherapeutic approaches (*d* = 1.12–2.67). However, the general results indicate that every treatment approach analyzed yielded significant effects.

However, the literature on treatment outcomes in IA is still both underdeveloped and heterogeneous in many ways, as it is also stated by the authors of the above-mentioned meta-analysis [24, page 327]: “*However this study illustrates the lack of methodological sound treatment studies, offers insight into the current state of internet addiction treatment research, bridges research investigations from “East” and “West” and is a first step in the development of an evidence-based treatment recommendation.*” This stresses the need for more clinical trials relying on accurately defined therapy programs. In the light of these circumstances, we will introduce a short-term psychotherapeutic treatment program for IA and provide first data from a pilot study regarding its usefulness and its effects. Although this pilot study may be based on a comparably small sample size and lacks inclusion of a wait-list control group, we regard it as helpful to publish these preliminary data.

### 1.1. Short-Term Treatment for Internet and Computer Game Addiction (STICA)

Since 2008, the work group of the Outpatient Clinic for Behavioral Addiction in Germany offered counseling for patients suffering from different kinds of IA. In the meantime, about 650 patients—mostly males aged between 16 and 35 years—introduced themselves as treatment seekers. In the light of increasing patient contacts, a standardized psychotherapeutic program for IA was developed and a therapy manual was developed (STICA) [25] which is based on cognitive-behavioral techniques known from treatment programs of other forms of addictive behavior. STICA is meant to be used for an outpatient treatment and consists of 15 group sessions plus additional eight sessions of individual therapy.

While the individual sessions are dealing with individual contents, the group sessions are following a clear thematic structure. In the first third of the program, the main themes approach the development of individual therapy aims, the identification of the Internet application that is associated with symptoms of IA, and the conduct of a holistic diagnostic investigation of psychopathological symptoms, deficits, resources, and comorbid disorders. Motivational techniques are also applied to enhance the patients' intention to cut down the dysfunctional behavior. In the second third, psychoeducative elements are introduced and deepened analyses of the Internet use behavior, focusing on its triggers and the reactions of the patient on cognitive, emotional, psychophysiological, and behavioral levels in that situation (SORKC-scheme, [18]), are performed. One crucial aim at this stage is the development of a personalized model of IA for each patient, based on the interaction of the Internet application used, predisposing and maintaining factors of the patient (e.g., personality traits) and the patients' social environment. At the last stage of the therapy, situations with heightened craving for getting online are further specified and strategies to prevent relapse are developed. A detailed overview on the structure of STICA is presented in Table 1.

### 1.2. Research Questions

In this study, we aimed at gathering first data on the effectiveness of STICA. We also intended to characterize the patients included regarding psychosocial symptoms, comorbidity, and personality features that can play a role in therapeutic treatment regarding building up a therapeutic alliance and differences in the treatment response [13]. Additionally, effects of psychosocial strain at the beginning of the therapy and personality traits on treatment outcome are reported. Lastly, we want to provide a comparison between patients regularly finishing the therapy (completers) and those who dropped out of the program (dropouts).

## 2. Materials and Methods

### 2.1. Data Acquisition and Statistical Analyses Plan

In this trial, data were collected from 42 patients consecutively introducing themselves to the Outpatient Clinic for Behavioral Addictions in Germany due to IA (clinical convenience sample). These patients were included out of an initial clinical sample of 218 treatment seekers. From these, 74 (33.9%) had to be excluded because of not meeting the criteria of IA. 29 (13.3%) more subjects had to be excluded because of being under the age of 17. 73 further exclusions (33.5%) were due to severe comorbid disorders, refusing to receive psychotherapeutic treatment, or severity of IA making an inpatient treatment necessary. The patients were asked to provide personal data for scientific processing and gave written informed consent. The investigation was in line with the declaration of Helsinki. Due to missing or incomplete data in the primary endpoints at T1, 5 subjects had to be excluded from the final data analyses.

Inclusion criteria were the presence of IA according to AICA-S (Scale for the Assessment of Internet and Computer Game Addiction, AICA-S [26]; see paragraph 2.2) and a standardized clinical interview of IA (AICA-C, Checklist for the Assessment of Internet and Computer Game Addiction, [15]). Moreover, male gender and age above 16 years were further requirements.

Exclusion criteria referred to severe comorbid disorders (other addictive disorders, psychotic disorders, major depression, borderline personality disorder, and antisocial personality disorder). Also, patients reporting current medication because of psychiatric disorders and those reporting being in psychotherapeutic treatment were excluded from data analyses.

As primary endpoints, the remission of IA according to a standardized self-report questionnaire (AICA-S) was defined. As secondary endpoints, changes in the following dimensional variables were assessed: severity of psychosocial symptoms, time spent online, negative consequences due to Internet use, and self-efficacy expectancy.

Data were assessed at the very beginning of the therapy (T0) and immediately after termination of the therapy (T1). Data analyses are reported for both conditions, intent to treat (including patients dropping out of treatment) and completers. For the intent-to-treat analyses, the last observation carried forward (LOCF) method was applied. The LOCF advises to use the last data available in those subjects not terminating a treatment condition regularly. In the present study, data from T0 were used for those subjects dropping out of the treatment program before T1 was assessed.

For statistical analyses, chi-square tests were used for the comparison of dichotomous variables with cramer-v as a measure of effect size. Changes in the primary and secondary endpoints were measured using paired *t*-tests for pre- and postcomparison for one sample, with *d*_*z*_ as a measure of effect size for dependent samples. According to the proposal by Dunlap et al. [27], adapted *d*_*z*_ was calculated if the correlation between the pre- and postscores of the dependent variables was bigger than 0.50. All analyses were performed using SPSS 21.

### 2.2. Instruments

For the classification of IA, two measures were applied at T0. For the Scale for the Assessment of Internet and Computer Game Addiction (AICA-S, [26]), a standardized self-report measure was applied assessing IA according to adapted criteria for gambling disorder and substance-related disorders (e.g., preoccupation, tolerance, withdrawal, and loss of control). Each criterion indicating IA is assessed either on a five-point Likert scale (never to very often) or in a dichotomous format (yes/no) and a weighted sum score can be derived from the accumulation of the diagnostic items. A cutoff of 7 points (that is corresponding to a total of 4 criteria which are met) has been found to have the best diagnostic accuracy in detecting IA (sensitivity = 80.5%; specificity = 82.4%) in an investigation of patients entering our outpatient clinic. According to previous investigations, AICA-S can be considered as showing good psychometric properties (Cronbach's *α* = .89), construct validity, and clinical sensitivity [11]. Since AICA-S was also the primary endpoint, it was also assessed at T1.

To further assure diagnosis of IA, a clinical expert rating was administered as well. The Checklist for Internet and Computer Game Addiction (AICA-C, [15]) was used for that purpose. AICA-C includes six core criteria for IA (preoccupation, loss of control, withdrawal, negative consequences, tolerance, and craving) that have to be rated by a trained expert on a six-point scale ranging from 0 = criterion not met to 5 = criterion fully met. According to analyses on its diagnostic accuracy, a cutoff of 13 points has yielded the best values (sensitivity = 85.1%; specificity = 87.5%). It has successfully been checked for its psychometric properties (Cronbach's *α* = .90) and its clinical accuracy [15].

The General Self-Efficacy Scale (GSE; [28]) was used in order to assess the construct of generalized self-efficacy expectancy by ten items. GES is understood as the quantity of subjective judgments of the amount of personal abilities to surmount problems and daily challenges. Numerous studies have reported that GSE has to be regarded as an important resilience factor, with high GSE predicting functional behavioral changes and motivating individuals to actively face engrossing situations [29]. GSE was administered at T0 and T1.

The NEO Five-Factor Inventory [30] was conceptualized to measure the five domains of the Five-Factor Model. It consists of 60 items answered on 5-point Likert scales and is one of the most used self-report measures in personality research. Numerous studies have stressed its good psychometric quality and validity [4]. The NEO-FFI was used only at T0 to examine predictive power of the five factors on therapy outcome and compliance.

At the measure points, T0 and T1, psychopathological symptoms were assessed using the Symptom Checklist 90R [31], a widely used clinical questionnaire with sound psychometric properties [32]. Psychopathological distress is assessed by 90 items (0 = no symptoms to 4 = strong symptoms) loading on nine subscales. The SCL-90R is referring to the degree to which the subject has experienced the symptoms during the last week. The global severity index (GSI)—a global sum score across the nine subscales—represents the overall distress.

## 3. Results

### 3.1. Description of the Sample

The sociodemographic statistics of the treatment seekers can be found in Table 2.

As can be derived from Table 2, most patients were not in a partnership with nearly the half of them still living at home with their parents. Most of the treatment seekers were not employed yet but owned a high school education.

Most of the patients were displaying addictive use of online-computer games (78.4%). 10.8% were using different Internet applications addictively, 8.1% used social networking sites, and 2.7% were doing excessive research in information data bases.

Regarding subclinical features, the following indices were found for NEO-FFI: *M* = 2.2 (SD = 0.60) for neuroticism, *M* = 1.9 (SD = 0.36) for extraversion, *M* = 2.4 (SD = 0.53) for openness, *M* = 2.3 (SD = 0.35) for agreeableness, and *M* = 1.8 (SD = 0.59) for conscientiousness.

### 3.2. Changes in Primary and Secondary Endpoints

70.3% (26) finished the therapy regularly (completers), 29.7% (11) patients dropped out during the course (dropouts). The results show that the completers had significant improvements in the primary and most of the secondary endpoints. The pre- and postscores of the primary and secondary endpoints for the completers can be derived from Table 3.

As can be seen in Table 3, significant decrease in the score of AICA-S is observable after the treatment. Moreover, significant decreases in hours spent online per weekend day and decreasing conflicts because of the Internet use in five of the six areas assessed were observable. Likewise, a significant decrease in the GSI was found, with completers displaying significantly diminished scores after treatment in seven of the nine subscales of the SCL-90R.

As expected, therapy effects were to some extent smaller when adding the dropouts to the analyses. However, the intent-to-treat analyses also reveal that after the treatment, the score in AICA-S decreased significantly (*t* = 6.09, *P* ≤ .001; *d*_*z*_ = 1.00). The same was observable for the average amount of time spent online at one day of the weekend (*t* = 5.48, *P* ≤ .001; *d*_*z*_ = .91) and overall negative consequences associated with the Internet use (*t* = 4.91, *P* ≤ .001; *d*_*z*_ = .81). Also, in psychopathological symptoms, significant pre- and postchanges were observable, regarding GSI (*t* = 4.39, *P* ≤ .001; *d*_*z*_ = .60) and the SCL-subscales obsessive-compulsive (*t* = 4.47, *P* ≤ .001; *d*_*z*_ = .66), social insecurity (*t* = 4.54, *P* ≤ .001; *d*_*z*_ = .46), depression (*t* = 4.37, *P* ≤ .001; *d*_*z*_ = .68), anxiety (*t* = 2.53, *P* ≤ .016; *d*_*z*_ = .39), aggression (*t* = 2.55, *P* ≤ .015; *d*_*z*_ = .42), phobic anxiety (*t* = 2.94, *P* ≤ .006; *d*_*z*_ = .35), and psychoticism (*t* = 4.18, *P* ≤ .001; *d*_*z*_ = .52). Also, the self-efficacy expectancy increased significantly after treatment (*t* = 4.99, *P* ≤ .001; *d*_*z*_ = .62).

### 3.3. Influence on Treatment Response

The analyses of sociodemographic differences between completers and dropouts showed no significant results regarding age, partnership, family status, living situation, or employment status. The only difference showing a trend significance (*χ* ^2^(3) = 6.38; *P* ≤ .055; cramer-v = .438) was found in education with completers displaying higher school education (76.9%) than dropouts (63.7%).

Regarding influence of personality traits on therapy termination, no significant group differences were found either, with the exception of the factor openness. A trend significance turned out indicating that completers (*M* = 2.5; SD = 0.53) were displaying higher scores than the dropouts (*M* = 2.2; SD = 0.47; *t* = 1.38, *P* = .082). Likewise, no group differences were found regarding psychosocial symptoms in T0 (SCL-90R) or the degree of self-efficacy expectancy (GSE). Also, severity of IA-symptoms did not discriminate between completers and dropouts nor did the amount of hours spent online (assessed by AICA-S).

## 4. Discussion

In this pilot study, we investigated effects of a standardized short-term psychotherapy on a sample of outpatient clients suffering from IA. To that purpose, a total of initially 42 patients were treated according to the therapy program with their psychological health status being assessed when entering the therapy and immediately after its termination. As a primary endpoint, we assessed symptoms of IA according to a reliable and valid self-report measure (AICA-S; [26]). Additionally, time spent online, negative consequences arising from online activities, self-efficacy expectancy, and psychosocial symptoms were defined as secondary endpoints.

About 70% of the treatment seekers passed the complete therapy program (completers), and about one-third dropped out during the course of the therapy. Thus, the dropout rate is well within the outpatient dropout rates within mental health care (see [33]; 19–51%) but exceeds those reported by Winkler and colleagues (see [24]; 18.6%). The further results indicate that the treatment program has promising effects. After the therapy, a significant decrease of IA-symptoms could be observed. The effects sizes found here amounted to *d*_*z*_ = 1.45 for the completers and *d*_*z*_ = 1.00 for the total sample including the dropouts. According to the definition of Cohen [34], this can be regarded as an indication of large effects. Moreover, it corresponds to the effect sizes on IA-status after psychotherapy (*d* = 1.48; with confidence intervals between .84 and 2.13) reported in the meta-analyses by Winkler et al. [24]. Likewise, time spent online on the weekends was significantly reduced after the therapy with a comparably large effect size (*d* = 1.31) that is nevertheless smaller compared to the data provided by the latest meta-analysis on that topic (see [24]; *d* = 2.38).

It is important to explain that the aim of this therapy approach is not to keep the patients away from any use of the Internet per se. Instead, specific therapy aims are developed based on the results of an extensive probatory in which Internet use habits of the patient are elucidated and problematically used Internet contents are identified. The therapy aims at motivating the patient to initiate abstinence from the Internet activity identified as being related to core symptoms of IA, like the loss of control and craving. Thus, a mean value of zero hours spent online was not expected. Indeed, the mean online time of 2.6 hours per day is well within the range of the German population average. In a representative survey on approximately 2500 German subjects, Müller et al. [35] reported that the average time spent online on a day of the weekend was 2.2 hours within regular Internet users.

Moreover, also most of the secondary endpoints changed significantly during the therapy. First of all, problems arising from addictive Internet use decreased in several areas, concerning frequency of family conflicts, denial of other recreational activities, frequency of health problems, struggles with friends, and negative effects on school or job performance. Self-efficacy expectancy increased with a medium effect size of *d* = .79 and the mean score in the GSE after treatment is comparable to the one derived from the general German population [28]. This indicates that optimistic expectancy towards the individual's ability to surmount appearing difficulties and challenges reaches an acceptable level after the treatment. If differences in self-efficacy expectancy among patients after treatment can be perceived as a predictor for middle- and long-lasting therapy, effects should be investigated in follow-up studies.

Lastly, psychosocial symptoms associated with IA decreased significantly after the treatment. This was the case for the global severity index as well as for seven of nine subscales of the SCL-90R. Large effect sizes were accomplished for the global severity Index and obsessive-compulsive and depressive symptoms, as well as for social insecurity.

Surprisingly, we did not find any variables distinguishing between patients passing the complete therapy and those dropping out of the program that could have served as valuable markers for the therapy success. There was a statistical trend indicating that patients with higher levels of educations were more likely to finish the therapy regularly. Also, we found—again as a trend—that patients completing the therapy display higher scores in the personality traits openness. In the personality literature, high openness is described as being interested in alternatives to traditional thinking and acting and showing curiosity towards new aspects and ways of thinking [36]. One might conclude from this that patients scoring high on this factor might have a more favorable attitude regarding psychotherapy and therefore are more likely to get themselves into the changes of psychotherapy. However, the relationships reported here were only marginally significant. This might be explained by the small sample size, especially concerning the patients dropping out of treatment. Clearly, more research is needed to identify predictors of therapy completion in patients with IA.

This study has a number of limitations that need to be addressed. A major shortcoming has to be seen in the lack of a control group, whether a wait-list control (WLC) or a therapy as usual group (TAU). Since there was only the single condition of a treatment group, statistical (by intraindividual comparisons) and interpretative limitations are obvious. It is not possible to finally determine if the effects of decreasing symptoms of IA and psychopathological strain are due to the psychotherapeutic intervention or origin from variables that were not controlled for. Secondly, a convenience sample of treatment seekers was examined without a randomization procedure. This raises the question if the participants of this study have to be regarded as selective. Moreover, the clinical sample under investigation was made up by only 42 male patients. This is quite a small sample size that did not allow for any deepened statistical analyses (e.g., the influence of different types of IA on therapy outcome). Since the sample was consisting only of male patients, the findings cannot be generalized to female patients. Lastly, the study design did not include a followup, so it is not possible to draw conclusions on the stability of the therapy effects that were observed immediately after the treatment. To correct these shortcomings, the authors are conducting a follow-up clinical trial at present [17]. This project that aims at the inclusion of 193 patients suffering from IA consists of a multicenter randomized and controlled trial with a follow-up assessment 12 months after the termination of the therapy.

## 5. Conclusion

Based on the data provided in this pilot study, it is reasonable to suppose that psychotherapeutic treatment of patients suffering from IA is effective. After application of a standardized cognitive-behavioral treatment, we found significant changes in symptoms of IA, time spent online, negative repercussions following the Internet use, and associated psychopathological symptoms, with the largest effects on depressive and obsessive-compulsive symptoms. This pilot study, which was conducted to herald the start of a larger, randomized, and controlled clinical trial, confirms the conclusions that Winkler and colleagues [24] have drawn from the data of their meta-analyses: IA appears to be a mental disorder that can be effectively treated by psychotherapeutic strategies—at least when referring to the immediate therapy effects.

## Figures and Tables

**Table 1:** Therapeutic elements of the therapy program “Short-term treatment for internet and computer game addiction” (STICA).

Probatory	Psychological diagnostic of IA
	Anamnesis of current and life-time media use
	Identification of problematically used online contents
	Diagnostic of comorbid disorders
	Assessment of psychopathological symptoms and psychosocial resources

Initial stage	Initializing trustful patient-therapist alliance and therapy commitment
	Assessment and enhancement of motivation for behavioral change
	Development of proximal and distal therapy aims
	Psychoeducation

Behavior modification	Psychoeducation
	Identification of triggers (situations, cognitions, beliefs, emotions, and psychophysiological reactions) of Internet use
	Development of SORKC-schemes and cognitive restructuring
	Development of an individual model of IA
	Training module: discrimination of emotional states
	Social skills training
	Reestablishment of alternative behavioral strategies and interests

Stabilization and relapse prevention	Reestablishment of alternative behavioral strategies and interests
	Exposure training and habituation training
	Individual relapse prevention, stopping techniques, and emergency plans

**Table 2:** Sociodemographic data of the treatment seekers included in this trial.

Sociodemographics	Treatment seekers *n* (%)
Gender (male)	37 (100%)
Age (*M*, SD)	26.1 (6.60)
Age (range)	18–47 years
Marital status	
Single	32 (86.5%)
Married	4 (10.8%)
Missing	1 (2.7%)
Partnership (no)	26 (70.3%)
Housing situation	
With parents	18 (48.6%)
Own household	13 (35.1%)
Shared living	6 (16.2%)
Education	
<10th grade	6 (16.2%)
>10th grade	27 (73.0%)
Still at school	4 (10.8%)
Employment status	
Employed (full-time/part-time)	8 (21.6%)
School/university/traineeship	21 (56.8%)
Unemployed	6 (16.2%)
Missing	2 (5.4%)

*n* = 37; *M* = mean, SD = standard deviation.

**Table 3:** Changes in primary and secondary endpoints in the completers.

Endpoint variable	Prescore completers	Postscore completers	Significance
Symptoms of IA (AICA-S)	12.1 (4.21)	3.5 (3.71)	*t* = 7.38, *P* = .001; *d*_*z*_ = 1.45
Hours spent online	7.6 (3.95)	2.6 (2.26)	*t* = 6.54, *P* = .001; *d*_*z*_ = 1.31
Problems (total)	3.7 (1.28)	1.5 (1.73)	*t* = 5.33; *P* = .001; *d*_*z*_ = 1.04
Problems in school/job	1.2 (1.14)	0.3 (0.45)	*t* = 4.91, *P* = .000; *d*_*z*_ = .96
Financial problems	0.1 (0.33)	0.1 (0.27)	Ns
Family conflicts	0.8 (0.40)	0.4 (0.50)	*t* = 4.28, *P* = .000; *d*_*z*_ = .84
Health problems	0.6 (0.50)	0.3 (0.45)	*t* = 3.14, *P* = .004; *d*_*z*_ = .62
Denial of recreational activities	0.8 (0.40)	0.4 (0.49)	*t* = 4.05, *P* = .001; *d*_*z*_ = .79
Conflicts with friends	0.7 (0.45)	0.2 (0.40)	*t* = 4.40, *P* = .001; *d*_*z*_ = 1.06
Self-efficacy expectancy	25.7 (4.24)	29.1 (4.38)	*t* = 5.42, *P* = .001; *d*_*z*_ = .79
SCL: GSI	0.7 (0.43)	0.3 (0.29)	*t* = 4.87, *P* = .001; *d*_*z*_ = .90
SCL: somatization	0.4 (0.38)	0.3 (0.30)	Ns
SCL: obsessive-compulsive	1.1 (0.70)	0.5 (0.49)	*t* = 4.81, *P* = .001; *d*_*z*_ = .91
SCL: social insecurity	0.8 (0.57)	0.4 (0.40)	*t* = 5.54, *P* = .001; *d*_*z*_ = .82
SCL: depression	1.1 (0.75)	0.5 (0.52)	*t* = 4.78, *P* = .001; *d*_*z*_ = .94
SCL: anxiousness	0.5 (0.44)	0.3 (0.25)	*t* = 2.61, *P* = .015; *d*_*z*_ = .51
SCL: aggressiveness	0.5 (0.54)	0.2 (0.33)	*t* = 2.71, *P* = .012; *d*_*z*_ = .53
SCL: phobic anxiety	0.3 (0.31)	0.1 (0.24)	*t* = 3.07, *P* = .005; *d*_*z*_ = .60
SCL: paranoid ideation	0.5 (0.49)	0.3 (0.39)	Ns
SCL: psychoticism	0.5 (0.46)	0.2 (0.24)	*t* = 4.52, *P* = .001; *d*_*z*_ = .67

*n* = 26.
